# A Review of Insomnia Treatments for Patients with Mild Cognitive Impairment

**DOI:** 10.14336/AD.2021.0423

**Published:** 2021-07-01

**Authors:** Meghan K Mattos, Angela Chang, Katherine Pitcher, Carley Whitt, Lee M Ritterband, Mark S Quigg

**Affiliations:** ^1^University of Virginia, School of Nursing, Charlottesville, VA 22903, USA.; ^2^Northwestern University Feinberg School of Medicine, Department of Medical Social Sciences, Chicago, IL 60611, USA.; ^3^University of Virginia Medical Center, Charlottesville, VA 22903, USA.; ^4^University of Virginia, School of Medicine, Charlottesville, VA 22903, USA.

**Keywords:** sleep, sleep initiation and maintenance disorders, cognitive dysfunction, cognitive behavioral therapy

## Abstract

Mild cognitive impairment (MCI) impacts approximately 20% of older adults, with many also experiencing sleep disorders, such as insomnia. Given the relationship between sleep and dementia, addressing sleep issues may offer an opportunity to treat reversible causes. There are two primary treatments for insomnia: behavioral-based (cognitive behavioral therapy for insomnia, CBT-I) and pharmacological interventions. Although CBT-I is recommended as first-line treatment for insomnia in older adults, sedative-hypnotics are more likely to be recommended than non-pharmacological treatments given their convenience and accessibility. However, there are significant concerns in prescribing medications to patients with MCI. To explore this disconnect, we reviewed insomnia treatments in older adults with MCI studies and current guidelines of pharmacological therapy. First, we reviewed studies presenting non-pharmacological treatment of insomnia in older adults with MCI. Although the search yielded over 4,000 non-duplicate titles, only one article presented data on non-pharmacological treatment of insomnia in MCI. The literature covering comorbid insomnia, CBT-I, and MCI is sparse. In contrast to review of non-pharmacological studies, studies on the pharmacological treatment of insomnia in older adults were ample. Finally, we reviewed international guidelines for pharmacological treatment of insomnia in cognitive disorders. More widely used pharmacological interventions show short-term effectiveness with problems of recurrence, ineffectiveness in inadvertent or purposeful chronic use, and adverse side effects. Despite evidence regarding adverse consequences, pharmacological treatment of insomnia remains the most common treatment for insomnia. Reflecting on age-related changes in older adults, particularly those with MCI, inappropriate or mismanagement of medication can lead to unnecessary complications. Further research examining effective behavioral-based sleep management options in older adults with cognitive impairment is needed with exploration of improved sleep on cognitive function.

Mild cognitive impairment (MCI, formerly “pre-dementia”) is an important transitional state between normal cognition and dementias, such as Alzheimer’s disease (AD) [1, 2]. MCI affects up to 20% of older adults and has an annual conversion to dementia of 8-15% [3, 4]. Sleep is an important factor in AD pathogenesis, where sleep disturbances and deprivation can increase levels of beta-amyloid and phosphorylated tau [5], a known cardinal pathophysiological feature of AD [6, 7], and lead to worse global cognition [8]. Individuals with AD pathology also have an increased inflammatory response, which increases beta-amyloid production [6, 9, 10]. Studies have shown that a lack of sleep likely contributes to the progression of AD [11] and may prevent interstitial fluid drainage through cerebrospinal fluid into interstitial space to clear beta-amyloid and phosphorylated tau from the brain [12, 13]. This increased beta-amyloid production is even more concerning as insomnia symptoms are known to negatively impact the brain’s ability to clear these beta-amyloid plaques [6, 7]. Although exact mechanisms are not yet known and may be bidirectional or interrelated [14, 15], sleep may mediate progression to AD, wherein sleep improvements could promote cognitive functioning.

Because insomnia is common in individuals with MCI, the evaluation and treatment of insomnia present an opportunity to intervene in the early stages of cognitive decline in disorders that generally present with no known cure. Current guidelines [16-20] recommend non-pharmacological cognitive-behavioral therapy for insomnia (CBT-I) as the first step in insomnia treatment with pharmacological intervention only offered if the CBT-I was not effective or available [20]. Based on the current guidelines and complications of polypharmacy in older adults with cognitive concerns, our original intention was to review current non-pharmacologic therapies in older adults with MCI, namely CBT-I. We found that these studies, quite simply, do not yet exist in a quantity that makes sense for a review. Therefore, we present a summary of selected studies and guidelines for this patient group’s prevalent pharmacological therapies. We argue that clinical trials of CBT-I in this at-risk elderly population are critically needed.

## Definitions, Incidence, and Comorbidities of Insomnia

Insomnia is a sleep disorder characterized by difficulty falling and/or staying asleep with impaired functioning resulting in daytime impairment [21]. Insomnia classification differs among the Diagnostic and Statistical Manual of Mental Disorders (DSM-V), the International Classification of Sleep Disorders (ICSD-2), and the International Classification of Disease (ICD-10, 2). These differences result in varying prevalence estimates, ranging from 5-50% across different definitions and populations [21, 23, 24]. Using the DSM-V, insomnia can be short-term (acute), where insomnia symptoms with impairment last one night to a few weeks, or chronic, where insomnia symptoms with impairment occur at least three times a week for at least three months [25]. Up to 50% of older adults report symptoms of insomnia that may or may not impact daytime functioning [21, 23]; however, chronic insomnia occurs in closer to 10-15% of the population [26, 27].

The burdens of insomnia are clear. Comorbid conditions may also be worsened by insomnia or through other bidirectional relationship(s) with comorbidities. Ninety-three percent of adults over 65 have at least one comorbid condition [28], which may help explain the higher rates of insomnia in this population [29]. The frequency and number of comorbidities may also contribute to inaccurate diagnosis, particularly when trying to differentiate between primary or comorbid insomnia. Common comorbid conditions in insomnia include depression, suicidal tendencies, heart disease, hypertension, myocardial infarction, diabetes, and metabolic syndrome [29]. Chronic insomnia affects physical, social, and emotional health [30]. Individuals with insomnia have decreased work productivity, more accidents, more hospitalizations, and overall greater healthcare costs than their healthy counterparts [31, 32]. For example, the cost of human capital loss due to insomnia is estimated to cost roughly $63 billion each year [33]. There are also significantly more direct costs for patients with insomnia compared to matched non-insomnia controls, including higher all-cause healthcare costs [34] and an estimated additional six-month direct medical expenditure of $1,143 [27]. Insomnia symptoms can also affect significant others and caregivers. Specifically, nighttime activities can disrupt partners’ sleep, which may impact their ability to function normally and provide care [35, 36].

## Insomnia and cognition

This review focuses on the comorbidities of cognition and insomnia in older adults [37-40]. Insomnia prevalence in patients with MCI or other cognitive disorders is as high as 50% (7). A recent systematic review found that insomnia was significantly associated with a 27% higher risk of cognitive disorders [41]. Another found a 2.14-fold increase in dementia risk in adults with primary insomnia [42]. Insomnia and cognition in the elderly may not only be co-associated; decreased attention and impaired memory of insomnia and sleep deprivation may present as dementia in older adults [35, 36, 43]. Whether confounding or comorbid to cognitive assessments, insomnia may have its own independent course, making treatment decisions in this population even more critical. Limited options are available for individuals with MCI to promote cognitive maintenance and prevent further cognitive decline. Intervention with effective insomnia treatments may provide an opportunity to intervene early and not only prevent, but potentially reverse, cognitive decline.

## Insomnia Treatments in Patients with MCI: A Disconnect between Recommendations and Evidence

Insomnia treatment is recommended in two forms: behavioral and pharmacological. The American Academy of Sleep Medicine [16], the British Association for Psychopharmacology [17], the American College of Physicians [18, 19], and the European Sleep Research Society [20] recommend CBT-I in the treatment of insomnia. Only when CBT-I is not effective or available should a pharmacological intervention be offered [20]. Traditional CBT-I targets maladaptive behaviors and dysfunctional thoughts that perpetuate sleep problems. Most clinicians conduct six to eight in-person, hour-long sessions and measure patient outcomes with subjective reports and daily sleep diaries. Across these sessions, there are typically five tenets presented as part of CBT-I: sleep restriction, stimulus control, cognitive restructuring, sleep hygiene, and relapse prevention. The focus of therapy is on establishing healthy sleep behaviors and reducing negative thoughts behaviors associated with sleep. Success is operationalized through demonstrated sustained symptom improvements, including SE, SOL, WASO, and TST [44]. This multi-modal approach allows for tailored, patient-centered treatment and promotes sustained symptom improvement.

The potential advantages of behavioral therapy include avoiding medication polypharmacy, medication dependence, and a focus on the underlying predisposing, precipitating, and/or perpetuating factors of chronic insomnia. Although non-pharmacological treatment is recommended as first-line treatment for insomnia in older adults, pharmacological interventions are widespread. We reviewed insomnia treatments in older adults with MCI studies and current guidelines of pharmacological therapy to explore this disconnect.

## Review of Non-Pharmacological Insomnia Treatment in Older Adults with MCI

A review was performed on non-pharmacological sleep interventions in older adults with MCI by searching PubMed, PyscInfo, and Web of Science through November 1, 2020. Search terms used were sleep, sleep-wake, circadian, insomnia, sleep initiation and maintenance disorders, sleep disturbance, mild cognitive impairment, cognitive dysfunction, and neuropsychiatric symptoms. Inclusion criteria included MCI diagnosis, intervention studies, human participants, and sleep disturbance with pre- and post-measurement. An MCI diagnosis required that participants undergo a neuropsychological assessment that included more than one assessment. For example, studies in which only the Mini-Mental Status Examination were administered as the neuropsychological assessment were excluded from this review. Exclusion criteria included subjective complaints of cognitive impairment for MCI diagnosis or diagnosis of Parkinson’s disease, traumatic brain injury, rapid eye movement sleep behavior disorder, spindle density, or multiple sclerosis. The outcome was cognitive status, as measured by at least one or more neuropsychological assessment(s). Most of the papers that did not meet inclusion or exclusion criteria evaluated a broad swath of sleep disturbances rather than insomnia specifically.

The qualifying article presented cognitive outcomes of an RCT of CBT-I in individuals with MCI [45, 46]. Patients (N=28) lived in two residential facilities. Participants were randomly assigned to CBT-I (n =14) or an active control group (n=14) that received a class on nutrition. The study intervention included four group sessions of CBT-I over eight weeks. Primary sleep outcomes were ?sleep latency (SL), wake after sleep onset (WASO), sleep efficiency (SE), and total sleep time (TST) measured by sleep diaries supplemented with actigraphy. The insomnia severity index (ISI), a highly validated subjective marker of insomnia, was also evaluated [47]. Secondary outcomes were measures of neuropsychological functioning including memory, verbal learning, attention, processing speed, executive functioning, task shift, and inhibition. All outcomes were assessed at three time points: baseline, post-intervention, and 4-month follow-up.

The CBT-I intervention group, compared with the control group, experienced improved SL, WASO, SE, and ISI at post-intervention and 4-month follow-ups [46]. Neuropsychological functioning findings demonstrated a statistically significant improvement in executive functioning based on the ?Delis Kaplan Executive Function System assessing inhibition (p<.05); however, there were no changes in the other neuropsychological domains, such as memory [45]. The authors concluded that in-person CBT-I for individuals with MCI is feasible, improves sleep outcomes, and may have long-term effects on cognition [46].

### Other Non-Pharmacological Treatments

Our review uncovered some non-pharmacological insomnia treatments that did not meet criteria, but, given the paucity of rigorous evidence for non-pharmacological therapies, bear mentioning. For example, one study of an alternative pharmacological therapy examined the use of a nutrient supplement comprised of omega-3 and omega-6 fatty acids plus pure γ-tocopherol [48] in an RCT of 46 older adults with probable MCI. We note that insomnia was not an entry criterion and that sleep was a secondary outcome. Significant improvements across ?memory, fluency, language, and the visuospatial cognitive domains were noted at 3- and 6-month follow-ups. Sleep subjectively improved in the treatment group. Light or acoustic therapies to improve cognition and sleep disorders in adults with dementia (rather than the present review’s criteria of MCI) have demonstrated mixed effectiveness in some measures of cognitive impairment and sleep physiology [49, 50]. For example, a recent pilot randomized crossover study presented the effect of a one-night acoustic versus sham stimulation on next-morning memory recall in patients with amnestic MCI and unknown insomnia status. Increasing slow-wave activity was associated with improved word recall, but larger efficacy studies are needed.

## Pharmacological Treatments for Insomnia in Patients With MCI

Our review’s striking finding is that only one therapy to improve insomnia symptoms individuals with MCI cohort qualified for discussion. This one reason is that pharmacological use remains so prevalent. We reiterate concerns over the use of sedative-hypnotic agents in elderly patients. Aging physiology heightens the risk of untoward side effects, drug interactions, and polypharmacy interactions. [51-53]. Age-related issues are especially concerning when using traditional benzodiazepines and non-benzodiazepine receptor agonist sleep aids such as zolpidem, eszopiclone, and zaleplon. The use of traditional sleep aids in older adults has been associated with serious adverse events, including cognitive impairment and falls [54, 55].

Industry-sponsored sedative-hypnotic trials in older adults, however, unexpectedly do not confirm these risks. Additionally, studies on the target population of MCI and insomnia are lacking. For example, a systematic review of pharmacotherapies for sleep disturbances (including but not limited to insomnia) in dementia found nine randomized controlled trials (RCTs) of medications including melatonin, trazodone, ramelteon, suvorexant, and lemborexant [56]. RCTs demonstrated significant but low- to moderate-effectiveness on sleep outcomes (such as sleep-diary measured sleep onset latency and actigraphy-measured sleep efficiency). RCTs demonstrated no higher serious adverse effects between intervention versus placebo arms. However, none examined the effects of improved sleep on cognition, and none included MCI as a cohort [56].

## Discussion

CBT-I is recommended as the first-line treatment for insomnia in older adults; however, in our review of non-pharmacological treatment of insomnia in MCI, we found studies are lacking. Most studies feature the use of sedative-hypnotics in the elderly or demented populations and focus on sleep effects rather than cognitive effects. The shortcomings in the literature in both treatment pathways are important in light of the proposed pathophysiology of Alzheimer’s dementia that state, overall, that sleep provides an important custodial function in brain health, and that disruptions of sleep lead to dementia [57, 58]. In this light, studies of sleep disorders of the at-risk population of patients with MCI to modify conversion to dementia appear critical. Our review findings that robust trials of improving insomnia - and hopefully preserving cognition - in patients with MCI are lacking.

Our review of CBT-I in MCI highlights this scarcity. We note that criteria for the diagnosis of MCI were inconsistent or insufficient across studies. Many studies required only one neuropsychological measure (e.g., memory) or a brief assessment tool, such as Mini Mental Status Examination (MMSE), for MCI diagnosis. These inclusion criteria do not measure up to clinical standards required to diagnosis MCI. Criteria for MCI, as defined by Petersen et al. (2001, 25), are regarded as the gold standard, and future studies including individuals with MCI should consider the use of comprehensive testing to promote generalizability of results.

One factor that may have limited studies is that non-pharmacological treatments for insomnia in MCI may be challenging to implement in those with later-stage dementia. Similar limitations applied to criteria to identify insomnia. Across sleep outcome studies, data collection methods ranged from self-report to lab-reported polysomnography. Sleep outcomes varied across studies, such that some emphasized sleep study measurements (sleep efficiency, wake after sleep onset, and sleep onset latency), and others focused on ISI. Another major limitation was that few studies specifically evaluated insomnia; many included sleep disturbances as a catch-all, less specific inclusion. The high prevalence of insomnia in patients with MCI and improved sleep’s potential effect on cognition warrants population-specific intervention studies.

Even though there are effective treatments for insomnia, less than 15% of adults with chronic insomnia are estimated to get treatment [60]. If a patient is interested in CBT-I and a provider recommends this first-line treatment, there are access barriers, including the limited number of clinicians trained in behavioral insomnia treatment [61]. However, one important development is the growth in the use of telemedicine and other online techniques, an aspect of medical care that has been emphasized by the medical system’s response to the novel coronavirus pandemic. Older adults have historically had limited Internet access, but this is changing dramatically. through government-funded broadband expansion [62] and the dramatic change in need for technological connections during the pandemic. Receiving therapy over the Internet may also allow patients with decreased attention [35, 36] to progress at their own pace, rather than the traditional once-a-week, one-hour, in-person sessions [63]. As an aside, cost of and access to CBT-I, at least in the US, is also a potential problem, that could be overcome using telemedicine or Internet-delivered CBT-I. Colleagues conducted a recent, definitive RCT of CBT-I delivery through the Internet showing effectiveness and long-term durability of effect [64]. A study evaluating the efficacy of a modified version for older adults (>55 years) with normal cognition is under analysis.

We recently conducted a pilot trial of Internet-delivered CBT-I in older adults with MCI and chronic insomnia. Older adults with MCI were recruited from hospital-based memory and sleep disorders clinics and enrolled in a single-arm intervention study. Participants completed the six-part CBT-I intervention over nine weeks with two-week pre- and post-assessments using self-report sleep diaries and wrist-worn actigraphs to obtain sleep measures over time. Participants with MCI demonstrated feasibility of Internet-delivered CBT-I and online sleep diary entry across baseline and post-intervention assessment periods [65]. Details and outcomes will be presented in a forthcoming publication that provides merit to conduct a Phase 2 trial with a larger sample size.

## Summary

There is limited research in patients with MCI using non-pharmacological, efficacious treatments. Pharmacological treatment may provide short-term improvements but can also come with adverse consequences. CBT-I is the recommended first-line treatment of insomnia in older adults and provides a personalized and less precarious option. However, insomnia intervention studies in older adults with MCI are lacking, perpetuating the disconnect between recommended first-line treatment and actual prescribing practice. Further research examining effective behavioral-based sleep management options in older adults with cognitive impairment are needed with a focused exploration of improved sleep on cognitive function.
